# Fiberoptic endoscopic assessment of dysphagia in a patient with cutaneous and oropharyngeal blisters

**DOI:** 10.1002/ccr3.8660

**Published:** 2024-03-14

**Authors:** Allison Hughes Bartholow

**Affiliations:** ^1^ Department of Speech Language Pathology UPMC Mercy Hospital Pittsburgh Pennsylvania USA

**Keywords:** blisters, deglutition, dysphagia, fiberoptic endoscopic evaluation of swallowing

## Abstract

Dysphagia resulting from bulbous pemphigoid is a rare but significant manifestation. A 77‐year‐old with bullous pemphigoid with no neurological history presented with severe oropharyngeal dysphagia, attributable to underlying blistering and edema, as documented on a fiberoptic endoscopic swallow examination.

## Graphical abstract

This case study provides fiberoptic endoscopic evidence of pharyngeal dysphagia in a patient with bullous pemphigoid.

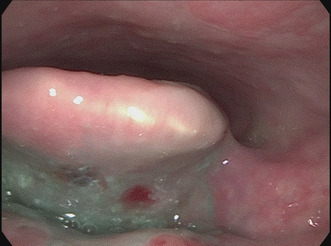

## 1 INTRODUCTION

Bullous pemphigoid is an autoimmune skin condition characterized by a nonbullous phase, with pruritic, erythematous patches, progressing to a bullous phase, with tense blisters and erythematous or urticarial plaques appearing on the limbs and lower trunk.^2^ Diagnosis involves direct immunofluorescence studies with evidence of IgG and/or C3.^3^ In total, 10%–30% of cases involve the mucosa in the oral, esophageal, and genital areas.^3^ Specifically, laryngeal mucosal involvement is reported in 4.9% of cases, with lesions often appearing along the laryngeal surface of the epiglottis. In those with laryngeal involvement, dysphagia is the primary reported symptom (43.8%).^4^ Case studies have reported progressive dysphagia and unintended weight loss with evidence of lesions and bleeding within the oropharynx, stenosis, strictures, and ulceration within the esophageal mucosa.^1^, ^3^, ^5^, ^6^, ^7^ To date, however, no endoscopic swallowing examination has been documented in the literature in this population. In this paper, we report a case of bullous pemphigoid with documented severe oropharyngeal dysphagia and characterize the subsequent endoscopic findings.

## 2 CASE HISTORY/EXAMINATION

A 77‐year‐old woman presented to the emergency department with 1 week of generalized weakness, reduced appetite, and unintended weight loss. Two years prior to her presentation, she was diagnosed with bullous pemphigoid, following a punch biopsy demonstrating subepidermal blistering, with minimal superficial perivascular and interstitial inflammatory infiltrate, composed of lymphocytes and very few eosinophils, with immunofluorescence studies showing linear deposits of IgG and C3 at the dermal‐epidermal junction.

She was subsequently treated with a combination of trimethoprim/sulfamethoxazole, doxycycline, and prednisone.

Her additional previous medical history included sleep apnea, chronic obstructive pulmonary disease, cholecystectomy, asthma, anticoagulation treatment for pulmonary embolism, pressure ulcers, hypothyroidism, Type 2 diabetes mellitus, generalized debility, ambulatory dysfunction, and gastrointestinal bleeding with multiple blood transfusions.

Of note, she reported a 2‐year history of odynophagia, globus sensation, and weight loss. Her prior swallowing history included completion of a Modified Barium Swallow Study (MBSS), 1 year previously, which showed severe oropharyngeal dysphagia characterized by poor lingual control, reduced hyolaryngeal excursion, diminished base of tongue retraction, reduced pharyngeal stripping wave, mistimed and incomplete laryngeal vestibule closure, and reduced upper esophageal sphincter opening. These deficits contributed to silent aspiration of thin, mildly thick, and moderately thick liquid consistencies and severe residue patterns with thick viscosities. Her origin of dysphagia was thought to be chronic in nature, given previous reports of a remote stroke, and therefore, a regular solid and thin liquid diet was recommended by the evaluating speech language pathologist. However, upon further review of her records, no cerebrovascular accident or acute neurological pathology had been documented.

She had an extensive 12 year gastroenterological history, with the completion of multiple procedures including upper gastroenterological series with kidney, ureters, and bladder (KUB), upper gastroenterological endoscopy, colonoscopy, upper esophageal ultrasound, multiple esophagoduodenoscopies, and Billroth‐II gastrojejunostomy, demonstrating an erythematous jejunum, oral cavity bleeding, and salmon‐colored mucosa, with suspected esophageal involvement secondary to bullous pemphigoid, and a small gastroenteric polypoid lesion with active bleeding. Additionally, she was followed by otolaryngology for hemolysis and oropharyngeal bleeding, with the completion of multiple flexible fiberoptic laryngoscopies prior to this admission, showing ulceration along the right buccal mucosa, and later, she developed a superficial lesion of the left soft palate. Current and prior brain imaging and chest X‐rays were also unremarkable; thus, neurological differential diagnoses for her dysphagia were ruled out.

## 3 METHODS

**FIGURE 1:**
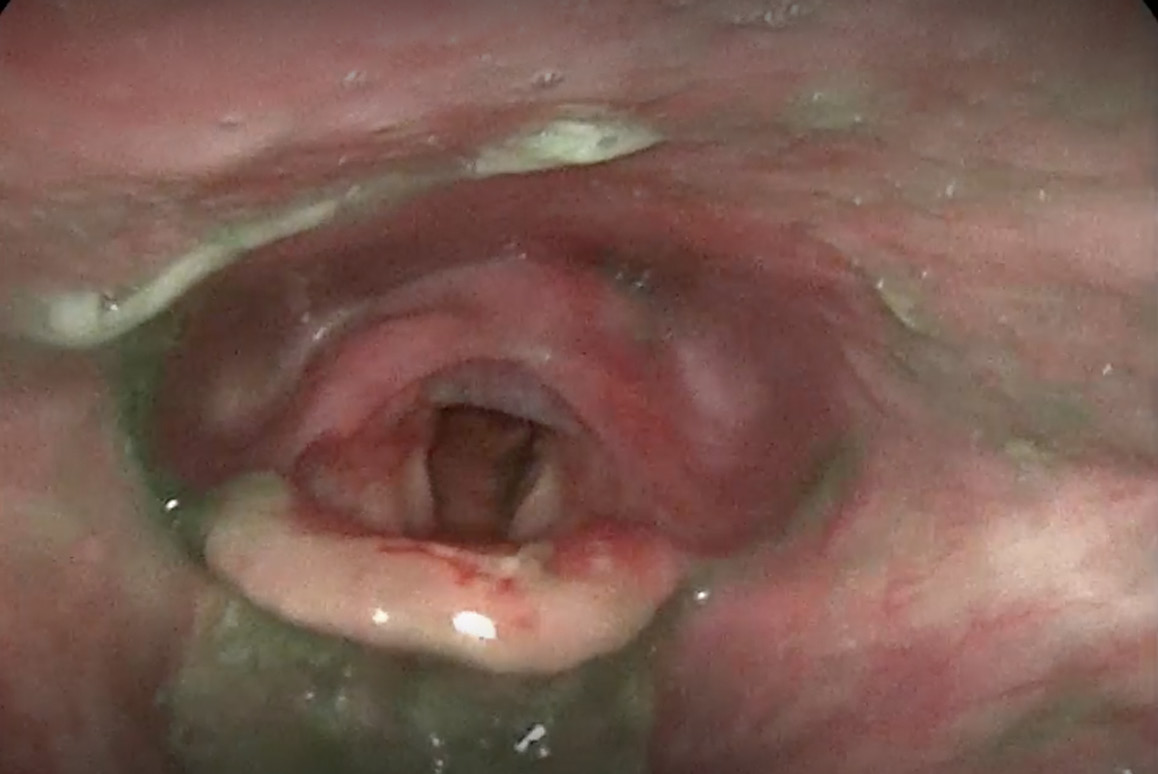
Evidence of silent tracheal aspiration and moderate residue patterns along the vallecular sinuses and the posterior pharyngeal wall following a single trial of thin liquids.

**FIGURE 2:**
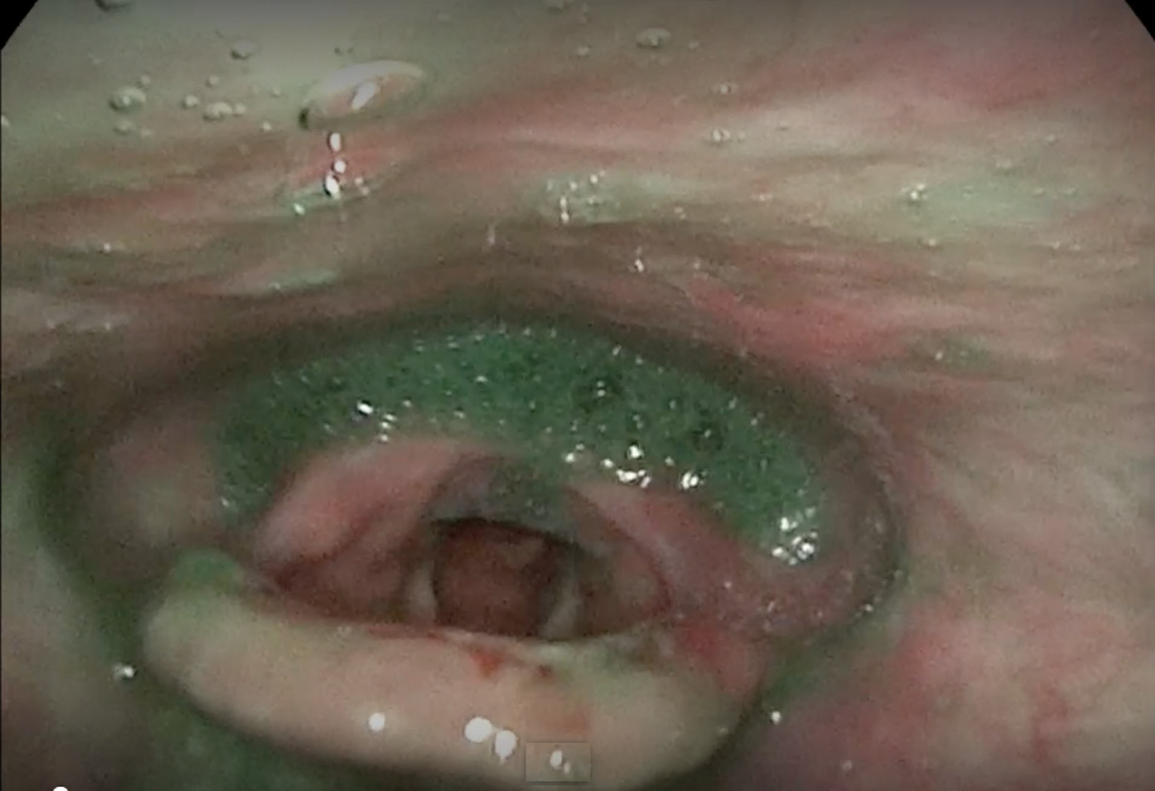
Esophageal backflow of green media from the region of the upper esophageal sphincter, which was misdirected into the airway.

**FIGURE 3:**
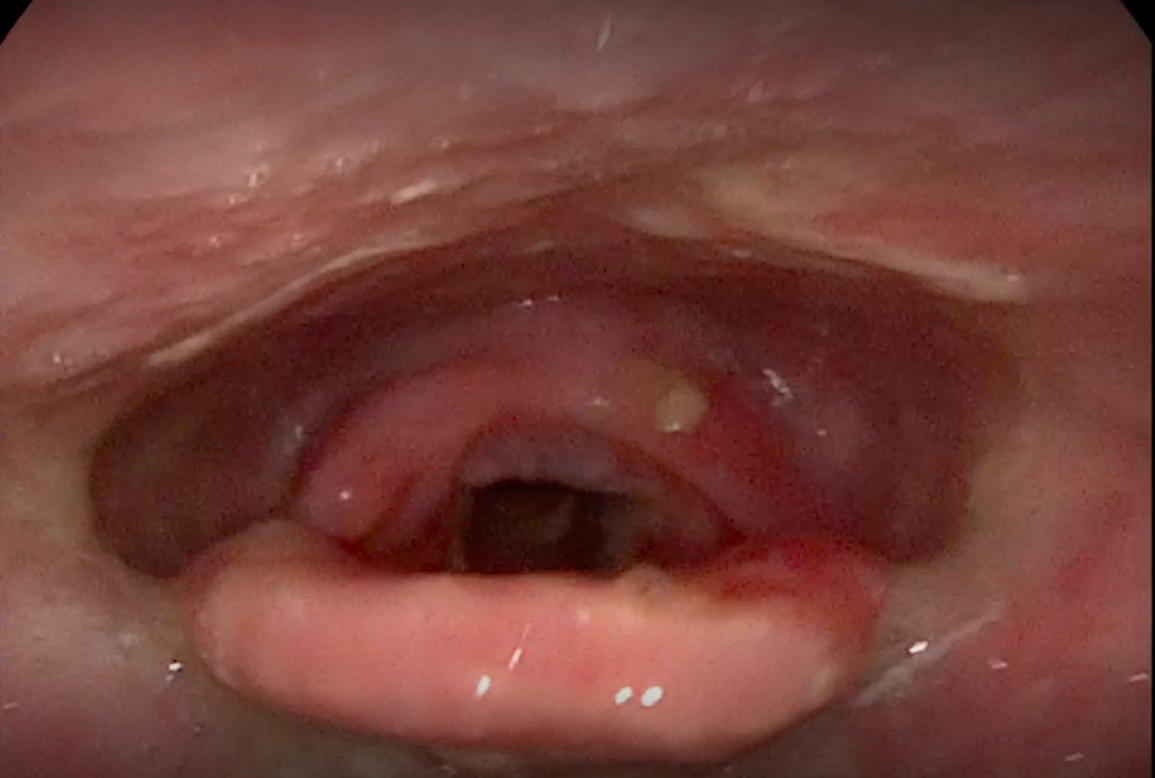
Mild edema observed along the arytenoids and posterior commissure, and erythema on the epiglottis, arytenoids, and pharyngeal wall.

**FIGURE 4:**
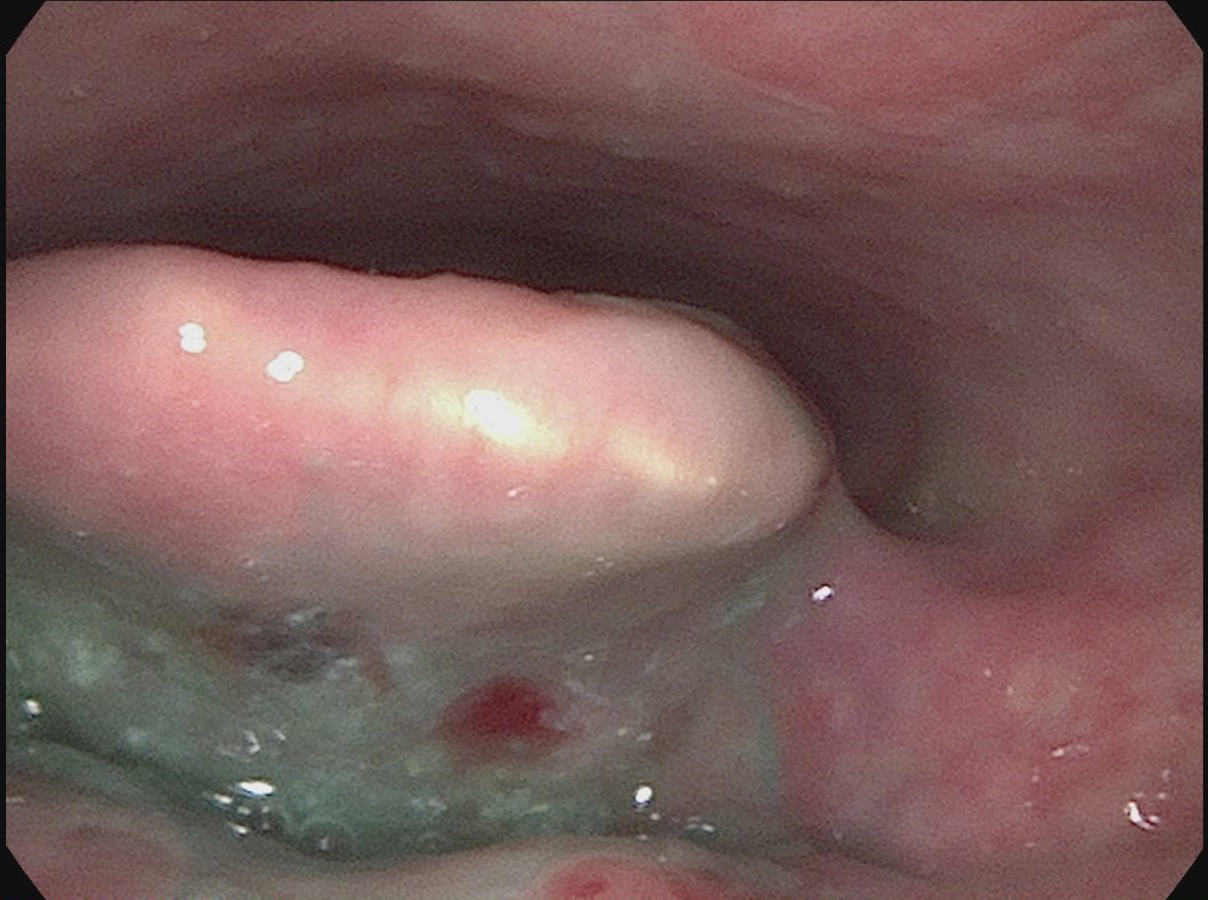
Red‐appearing blister within the vallecular sinus.

Speech language pathology completed an initial clinical swallow evaluation 4 days post admission. She presented with open and bleeding blisters along the upper and lower extremities, buttocks, and back. An oral mechanism examination was unremarkable for cranial nerve deficits. Red‐appearing ulcers were located along the buccal mucosa within the oral cavity, and there was erythema along the faucial arches. There were no overt signs or symptoms of aspiration on trials of ice chips, thin water, pureed solids, and coarse solids; however, she reported a globus sensation with solid trials. Given her extensive history, a Fiberoptic Endoscopic Evaluation of Swallowing (FEES) was recommended, which was completed 2 days later. The results showed incomplete whiteout, which indicated reduced pharyngeal constriction and suspected reduced hyolaryngeal excursion which contributed to moderate residue patterns in the vallecular and pyriform sinuses (Figure 1). Additionally, there was evidence of both silent and sensate aspiration of all trials with green and blue tinged media present within the laryngeal vestibule and subglottis immediately following white out (Figure 1). Retrograde flow from the upper esophageal sphincter occurred in between swallows which spilled into the airway, resulting in aspiration (Figure 2). Given the frequency, depth, and amount of airway invasion and diffuse residue patterns, the overall swallow function appeared to be indicative of severe dysfunction. Diffuse edema and erythema were observed throughout the pharynx and larynx (Figure 3). A blister was present within the vallecular sinus (Figure 4). Overall, her swallowing pathophysiology was consistent with her prior modified barium swallow examination (MBS). She was recommended to remain NPO, given the severity of her swallow function. After a discussion with her physicians, she ultimately elected to resume consuming a regular solid and thin liquid diet, after accepting the risk of aspiration.

## 4 DISCUSSION

This patient presented with severe oropharyngeal dysphagia with mixed sensate aspiration, severe residue patterns, and significant reflux patterns. Given the absence of neurological pathology, with unremarkable brain imaging, as well as anatomic evidence of a blister within the pharynx and diffuse edema and erythema, it can be theorized that the etiology of the patient's dysphagia is her underlying bullous pemphigoid. A small number of case reports have utilized flexible nasendoscopy, laryngoscopy, esophagogastroduodenoscopy, and esophagram studies to examine the oropharynx, larynx, and esophagus. However, to date, no research studies have directly assessed oropharyngeal swallowing function through either fiberoptic endoscopic evaluation of swallowing (FEES) or modified barium swallow (MBS) examination in this specific population. Prior studies suggest lesions related to bullous pemphigoid within the oropharynx, larynx, and esophageal mucosa may contribute to odynophagia, scarring, stenosis, and active bleeding, leading to the development of oropharyngeal dysphagia; however, none have directly evaluated the act of swallowing through dynamic instrumental swallow examination.^1^, ^2^, ^3^, ^4^, ^5^, ^6^, ^7^, ^8^ This unique case study provides novel insight into and endoscopic documentation of the potential pathophysiology of swallowing in cases of bullous pemphigoid.

## 5 CONCLUSION AND RESULTS

In summary, this paper describes a patient with bullous pemphigoid without significant neurological history, who presented with weight loss, globus sensation, and odynophagia, and was later found to have severe oropharyngeal dysphagia with physical evidence of pharyngeal bullae based upon a fiberoptic endoscopic evaluation of swallowing (FEES), which is, to our knowledge, the first time such an assessment tool to study dysphagia in the bullous pemphigoid population has been documented. Many studies have suggested impairment of, but have not directly measured, swallowing function in a patient with bullous pemphigoid. The key clinical message is in the absence of other neurological diagnoses, bullous pemphigoid may lead to development of significant oropharyngeal and esophageal dysphagia in individuals who present with bullae in the oropharynx or larynx, and fiberoptic endoscopic evaluation of swallowing (FEES) is a novel and useful instrumental assessment tool to directly evaluate suspected dysphagia in such cases.

## AUTHOR CONTRIBUTIONS

**Allison Hughes Bartholow:** Conceptualization; methodology; project administration; writing – original draft; writing – review and editing.

## FUNDING INFORMATION

None.

## CONFLICT OF INTEREST STATEMENT

None to report.

## CONSENT

Written informed consent was obtained from the patient to publish this report in accordance with the journal's patient consent policy.

## Data Availability

The author confirms that the data supporting the findings of this study are available within the article and its supplementary materials.
