# Correction to “Fiberoptic Endoscopic Assessment of Dysphagia in a Patient With Cutaneous and Oropharyngeal Blisters”

**DOI:** 10.1002/ccr3.70196

**Published:** 2025-02-17

Bartholow AH. Fiberoptic endoscopic assessment of dysphagia in a patient with cutaneous and oropharyngeal blisters. *Clin Case Rep*. 2024; 12:e8660. doi:10.1002/ccr3.8660

In the abstract of the article, the first line reads “dysphagia resulting from bulbous pemphigoid is a rare but significant manifestation.” The word “bulbous” is incorrect. The word “bulbous” needs to be replaced with “bullous.” The first line should have read “dysphagia resulting from bullous pemphigoid is a rare but significant manifestation.”

We apologize for this error.

